# Supplementary material for: Splice-Junction-Based Mapping of Alternative Isoforms in the Human Proteome
Source: Cell Rep. Author manuscript; Available in PMC 2020 Jan 15. (PMC6961840; doi:10.1016/j.celrep.2019.11.026)
Supplement: 2 [file NIHMS1546469-supplement-2.zip › DF1/PXD010154/Thyroid/Thyroid_48_ANKRD13D_ILQLSLTEPR.pdf]

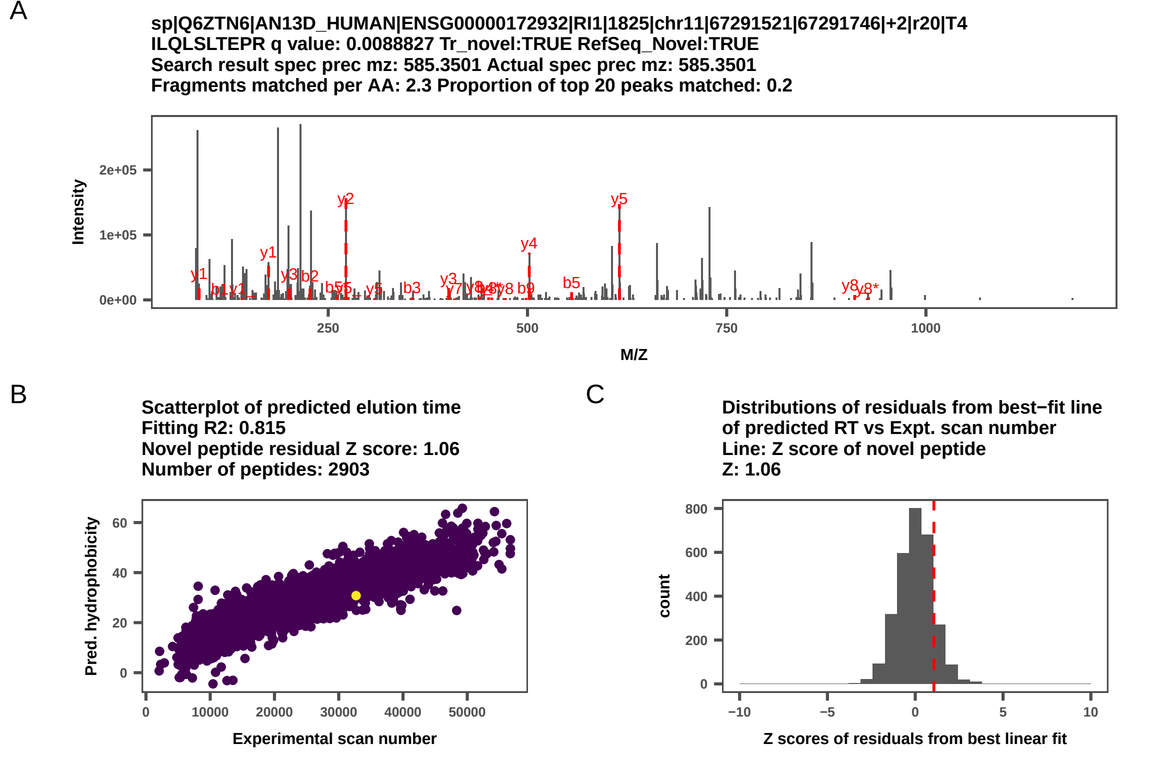
A
sp|Q6ZTN6|AN13D_HUMAN|ENSG00000172932|RI1|1825|chr11|67291521|67291746|+2|r20|T4
ILQLSLTEPR q value: 0.0088827 Tr_novel:TRUE RefSeq_Novel:TRUE
Search result spec prec mz: 585.3501 Actual spec prec mz: 585.3501
Fragments matched per AA: 2.3 Proportion of top 20 peaks matched: 0.2
Intensity
2e+05
1e+05
0e+00
y2
y5
y4
y1
b5
y8
250
500
750
1000
M/Z
B
Scatterplot of predicted elution time
Fitting R2: 0.815
Novel peptide residual Z score: 1.06
Number of peptides: 2903
Pred. hydrophobicity
60
40
20
0
0
10000
20000
30000
40000
50000
Experimental scan number
C
Distributions of residuals from best−fit line
of predicted RT vs Expt. scan number
Line: Z score of novel peptide
Z: 1.06
count
800
600
400
200
0
−10
−5
0
5
10
Z scores of residuals from best linear fit
